# Mechanism of bombesin-induced tonic contraction of the porcine lower esophageal sphincter

**DOI:** 10.1038/srep15879

**Published:** 2015-11-02

**Authors:** Ching-Chung Tsai, Li-Ching Chang, Kai-Jen Lin, Shu-Leei Tey, Yu-Tsun Su, Ching-Wen Liu, Tong-Rong Tsai, Shih-Che Huang

**Affiliations:** 1 School of Pharmacy, Kaohsiung Medical University, Kaohsiung, Taiwan, No. 100, Shih-Chuan 1st Road, Sanmin District, Kaohsiung City, Taiwan, R.O.C; 2 Department of Pediatrics, E-Da Hospital, I-Shou University, Kaohsiung, Taiwan, No. 1, Yi-Da Road, Yan-Chao, Kaohsiung City, Taiwan, R.O.C; 3 Department of Occupational Therapy, I-Shou University, Kaohsiung, Taiwan, No. 8, Yi-Da Road, Yan-Chao, Kaohsiung City, Taiwan, R.O.C; 4 Department of Pharmacy, E-Da Hospital, I-Shou University, Kaohsiung, Taiwan, No. 1, Yi-Da Road, Yan-Chao, Kaohsiung City, Taiwan, R.O.C; 5 Department of Pathology, E-Da Hospital, I-Shou University, Kaohsiung, Taiwan, No. 1, Yi-Da Road, Yan-Chao, Kaohsiung City, Taiwan, R.O.C; 6 Department of Internal Medicine, E-Da Hospital, I-Shou University, Kaohsiung, Taiwan, No. 1, Yi-Da Road, Yan-Chao, Kaohsiung City, Taiwan, R.O.C; 7 School of Medicine, I-Shou University, Kaohsiung, Taiwan, No. 8, Yi-Da Road, Yanchao, Kaohsiung City, Taiwan, R.O.C

## Abstract

Gastroesophageal reflux disease (GERD) is a disorder that is related to an incompetent lower esophageal sphincter (LES). Previous studies showed that bombesin could increase LES pressure in humans and opossums. The aim of the present study was to characterize the effects of bombesin on porcine LES contraction. We used the selective agonists, neuromedin B (NMB), gastrin-releasing peptide (GRP), and [D-Tyr^6^,Apa-4Cl^11^,Phe^13^,Nle^14^]bombesin-(6-14) (DTACPN-BN), as well as receptor antagonists of bombesin receptor subtype 2 (BB_2_), and 3 (BB_3_) for *ex vivo* contraction studies. Atropine, nifedipine, tetrodotoxin, and ω-conotoxin GVIA were used to explore the agonist-induced LES contraction mechanism. Reverse transcription polymerase chain reaction and immunohistochemistry were applied to detect bombesin receptor expression. Our results indicate that GRP and DTACPN-BN, but not NMB, induced tonic contractions of the porcine LES in a dose-dependent manner, and the contractions were inhibited with selective BB_2_ and BB_3_ antagonists. The GRP-induced contraction is mainly caused by L-type Ca^2+^ channel-mediated Ca^2+^ influx. However, DTACPN-BN-induced contractions are associated with neuronal conduction. RT-PCR and immunohistochemistry revealed that BB_2_ and BB_3_ were expressed in the porcine LES. Bombesin-induced tonic contraction of the LES is mediated through BB_2_ and BB_3_. Bombesin, BB_2,_ and BB_3_ agonists might have the potential to treat GERD.

Gastroesophageal reflux disease (GERD) is caused by acid- or other irritant-induced injury from stomach backflow to the esophagus. Under physiological conditions, the lower esophageal sphincter (LES) is closed to prevent damage from food or acid reflux and transiently relaxes to let food pass through when swallowing occurs. The main underlying mechanism of GERD is LES incompetence, which may result from decreased LES tone or recurrent inappropriate transient LES relaxations. An incompetent LES cannot prevent acid reflux, which in turn results in damage to the esophageal mucosa, which further diminishes the LES pressure. GERD management includes lifestyle and dietary modifications, medications, which include antacids, histamine 2 receptor antagonists, and proton pump inhibitors (PPI), and surgery^1^. PPI is the main first-line drug for GERD treatment, but 10% to 40% of patients are refractory to PPI therapy^2^. Additionally, long-term PPI use may cause some side effects, such as micronutrient absorption, drug interference and microscopic colitis^3^. Surgical procedures, such as Nissen fundoplication, increase the LES resting tone and can lead to an improved esophageal contraction amplitude^4^. However, symptom recurrence may occur after fundoplication. Medications to strengthen the lower esophageal sphincter, such as baclofen, which reduces transient LES relaxation, are few in number. Baclofen may cause severe neurological side effects, such as weakness, confusion, tremors, and dyskinesia^5^. Therefore, drugs that enhance LES contractions in order to avoid acid or other irritant reflux are being developed.

Bombesin is a 14-amino acid peptide and originates from the skin of the European fire-bellied toad (*Bombina bombina*)^6^,^7^. There are three bombesin receptor subtypes, including bombesin receptor subtype 1 (BB_1_), bombesin receptor subtype 2 (BB_2_), and bombesin receptor subtype 3 (BB_3_)^7^,^8^. Bombesin can stimulate G cells in the stomach to release gastrin. Bombesin is also considered to be involved in satiety and appetite regulation, as well as in stress-induced anorexia and pressure-induced obesity^9^,^10^. These phenomena may be related to a specific distribution of bombesin receptors in the edge region of the cerebral cortex^11^. Furthermore, an increase in bombesin receptor activity may be indicative of a number of tumors, such as small cell lung cancer, gastric cancer, and neuroblastoma^12^.

Bombesin is a potent LES contractile agent in opossums. The contraction mechanism involves direct effects on smooth muscle and indirect postganglionic neuron stimulation^13^. Bombesin increases the LES pressure in humans and can affect esophageal motility by increasing peristaltic wave amplitude and duration. Neither the effects of vagal cholinergic agents nor the release of gastrointestinal hormones caused by bombesin are considered to be related to the mechanism of action of bombesin on esophageal motility in humans^14^.

Although previous studies found that bombesin can increase LES contraction in opossums and LES pressure in humans, the bombesin receptor subtypes involved and the underlying mechanism are still not clear^13^,^14^. Here, we used porcine LES to investigate which of the bombesin receptor subtypes, BB_1_, BB_2_, and BB_3_, are involved in bombesin-induced LES contraction. The results of this study support the bombesin drug potential in GERD treatment.

## Results

### The effects of bombesin and the bombesin receptor agonists on porcine LES sling and clasp muscle strip tension

**Figure 1:**
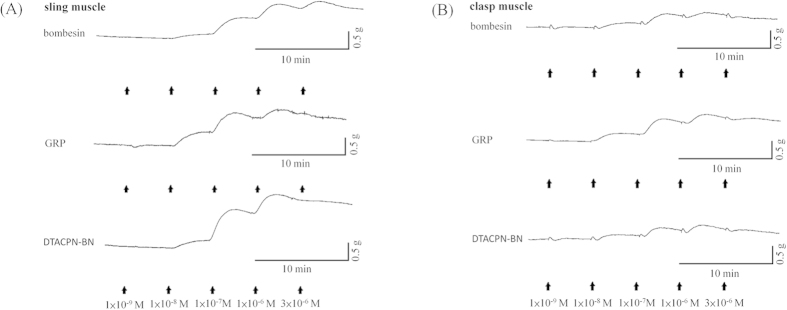
Typical contraction tracings of the porcine LES sling (A) and clasp (B) muscle strips that were caused by bombesin, gastrin-releasing peptide (GRP), and [D-Tyr^6^,Apa-4Cl^11^,Phe^13^,Nle^14^]bombesin-(6-14) (DTACPN-BN). The arrows indicate the addition of bombesin, GRP and DTACPN-BN in cumulative doses at 3 min intervals.

**Figure 2:**
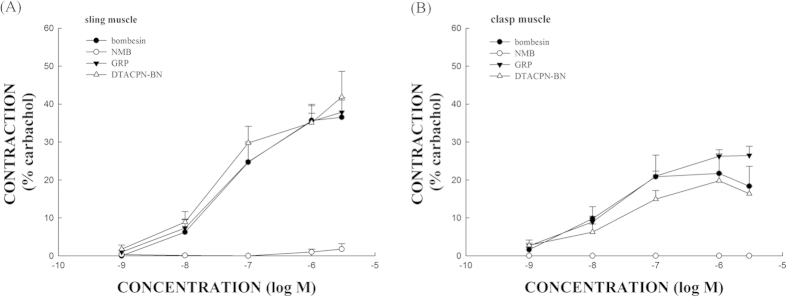
The ability of bombesin, neuromedin B (NMB), gastrin-releasing peptide (GRP), and [D-Tyr^6^,Apa-4Cl^11^,Phe^13^,Nle^14^]bombesin-(6-14) (DTACPN-BN) to cause porcine LES sling (A) and clasp (B) muscle strip contractions. The values are expressed as a percent of a carbachol (1 μM)-induced contraction. The results given are from at least four experiments. The vertical bars represent ± standard error of the mean (SEM).

The structure of the porcine LES is similar to that of the human LES. It is composed of sling muscle fibers at the greater curvature (GC) and clasp muscle fibers at the lesser curvature (LC). Bombesin receptor agonists including a BB_1_ agonist, neuromedin B (NMB), a BB_2_ agonist, gastrin-releasing peptide (GRP), and a BB_3_ agonist, [D-Tyr^6^,Apa-4Cl^11^,Phe^13^,Nle^14^]bombesin-(6-14) (DTACPN-BN), were used to elucidate which bombesin receptor subtype or subtypes are involved in bombesin-induced LES contraction. Bombesin, GRP, and DTACPN-BN all elicited sustained and dose-dependent contraction of the isolated porcine LES sling and clasp muscle strip (Fig. 1A,B). Figure 2A shows that bombesin caused detectable contraction of the LES sling muscle strips at 1 nM and maximal contraction at 3 μM. The maximal tension caused by 3 μM bombesin was 36 ± 5% of the tension caused by 1 μM carbachol (n = 4). In the LES clasp muscle strips, bombesin caused detectable contraction at 1 nM and maximal contraction at 1 μM (Fig. 2B). The maximal tension caused by 1 μM bombesin was 22 ± 5% of the tension caused by 1 μM carbachol (n = 4). Similarly, GRP caused detectable contraction of the LES sling muscle strips at 1 nM, half-maximal contraction at 0.18 ± 0.04 μM, and maximal contraction at 3 μM (Fig. 2A). The maximal tension caused by 3 μM GRP was 38 ± 4% of the tension caused by 1 μM carbachol (n = 8). In the LES clasp muscle strips, GRP caused detectable contraction at 1 nM, half-maximal contraction at 42 ± 10 nM, and maximal contraction at 3 μM (Fig. 2B). The maximal tension caused by 3 μM GRP was 26 ± 2% of the tension caused by 1 μM carbachol (n = 7). DTACPN-BN, furthermore, caused detectable contraction of the LES sling muscle strips at 1 nM and maximal contraction at 3 μM (Fig. 2A). The maximal tension caused by 3 μM DTACPN-BN was 42 ± 7% of the tension caused by 1 μM carbachol (n = 4). In the LES clasp muscle strips, DTACPN-BN caused detectable contraction at 1 nM and maximal contraction at 3 μM (Fig. 2B). The maximal tension caused by 1 μM DTACPN-BN was 20 ± 2% of the tension caused by 1 μM carbachol (n = 4). Both DTACPN-BN and GRP induced dose-dependent tonic contractions in the isolated porcine LES sling and clasp muscle strips. In contrast, NMB did not induce a contraction (Fig. 2A,B).

### The effects of BB_2_ and BB_3_ antagonists on GRP- and DTACPN-BN-induced porcine LES sling and clasp muscle strip contractions

**Figure 3:**
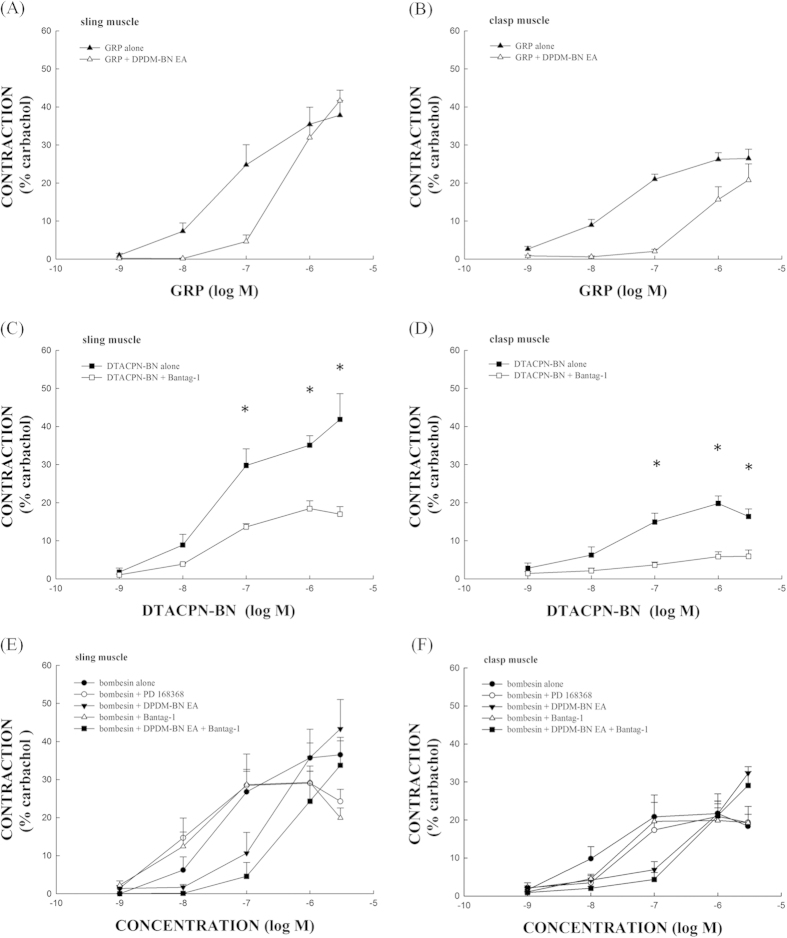
The effects of bombesin receptor subtype 1 (BB_1_), 2 (BB_2_) and 3 (BB_3_) antagonists on gastrin-releasing peptide (GRP)-, [D-Tyr^6^,Apa-4Cl^11^,Phe^13^,Nle^14^]bombesin-(6-14) (DTACPN-BN)-, and bombesin-induced contractions of the porcine LES sling and clasp muscle strips. The BB_2_ antagonist, [D-Phe^6^,des-Met^14^]BN(6-14) ethylamide (DPDM-BN EA) (1 μM), had a significant inhibitory effect on LES sling (**A**) and clasp (**B**) muscle strip contractions that were induced by GRP. The EC_50_ differences between the samples treated with GRP alone and with GRP plus DPDM-BN EA were significant (*p* < 0.05). The BB_3_ antagonist, Bantag-1 (1 μM), significantly inhibited the DTACPN-BN-induced LES sling (**C**) and clasp (**D**) muscle strip contractions (*p* < 0.05). *Represents significant differences compared with the DTACPN-BN alone (*p* < 0.05). Only DPDM-BN EA (1 μM) plus Bantag-1 (1 μM) had a significant inhibitory effect on LES sling (**E**) and clasp (**F**) muscle strip contractions that were induced by bombesin. The EC_50_ difference between the levels of bombesin alone and the levels of bombesin plus DPDM-BN EA combining with Bantag-1 was significant (*p* < 0.05).

A BB_2_ antagonist, [D-Phe^6^,des-Met^14^]BN(6-14) ethylamide (DPDM-BN EA) and a BB_3_ antagonist, Bantag-1, were used to block GRP- and DTACPN-BN-induced porcine LES contraction, respectively. Incubation with DPDM-BN EA (1 μM) shifted the GRP concentration-response curve to the right, with three- and fourteen-fold changes at the EC_50_ levels for the LES sling (Fig.3A) and clasp (Fig. 3B) muscle strips, respectively. The GRP alone EC_50_ levels were 0.18 ± 0.04 μM (sling) and 42 ± 10 nM (clasp) (n = 4/group). The GRP plus 1 μM DPDM-BN EA EC_50_ levels were 0.58 ± 0.10 μM (sling) and 0.61 ± 0.07 μM (clasp) (p < 0.05, compared with GRP alone, respectively; n = 4/group). These results show that DPDM-BN EA had a significant inhibitory effect on GRP-induced contraction of both the porcine LES sling and clasp muscle strips. Similarly, Bantag-1 significantly inhibited DTACPN-BN-induced contraction of the porcine LES sling and clasp muscle strips (*p* < 0.05, n = 4/group) at a concentration of 1 μM (Fig. 3C,D).

### The effects of BB_1_, BB_2_ and BB_3_ antagonists on bombesin-induced porcine LES sling and clasp muscle strip contractions

To further examine whether the bombesin-induced LES contraction is mediated through BB_1,_ BB_2_ and/or BB_3_, LES muscle strips were treated with BB_1_ (PD 168368), BB_2_ and BB_3_ antagonists, respectively, before the bombesin stimulation. PD 168368 (1 μM) and Bantag-1 (1 μM) alone did not inhibit bombesin-induced contraction of both the porcine LES sling (Fig. 3E) and clasp (Fig. 3F) muscle strips (p > 0.05, n ≥ 4/group). However, the BB_2_ antagonist DPDM-BN EA (1 μM) shifted the bombesin concentration-response curve to the right but the difference between the EC_50_ levels of bombesin alone and EC_50_ levels of bombesin plus DPDM-BN EA was not significant (p > 0.05, n = 4/group). In contrast, the BB_2_ antagonist DPDM-BN EA combining with BB_3_ antagonist Bantag-1 (both 1 μM) significantly shifted the bombesin concentration-response curve to the right, with ten- and sixty-fold changes at the EC_50_ levels for the LES sling and clasp muscle strips, respectively. Specifically, the EC_50_ levels of bombesin alone were 72 ± 21 nM (sling) and 10 ± 0.51 nM (clasp) (n = 4/group). The EC_50_ levels of bombesin plus 1 μM DPDM-BN EA combining with Bantag-1 were 0.72 ± 0.22 μM (sling) and 0.69 ± 0.14 μM (clasp), respectively (both p < 0.05, compared with bombesin alone; n = 6 and 5/group, respectively). Thus, it is likely that the bombesin-induced LES contraction is mediated through BB_2_ and BB_3_.

### The effects of tetrodotoxin (TTX), atropine, and nifedipine on GRP-induced porcine LES sling muscle strip contractions

**Figure 4:**
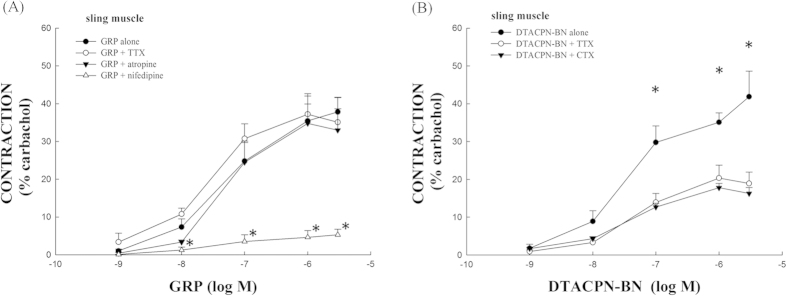
The effects of tetrodotoxin (TTX), atropine, and nifedipine on gastrin-releasing peptide (GRP)-induced LES sling muscle contractions and the effects of ω-conotoxin GVIA (CTX) and tetrodotoxin (TTX) on LES sling muscle contractions induced by [D-Tyr^6^,Apa-4Cl^11^,Phe^13^,Nle^14^]bombesin-(6-14) (DTACPN-BN). (**A**) TTX (1 μM) and atropine (1 μM) had no significant effects on LES sling muscle strip contractions that were induced by GRP. In contrast, nifedipine (1 μM) had a significant inhibitory effect on LES sling muscle strip contractions that were induced by GRP. *Represents significant differences compared with GRP alone (*p* < 0.05). (**B**) Both CTX and TTX significantly (*p* < 0.05) inhibited the DTACPN-BN-induced porcine LES sling muscle fiber contractions. *Significantly different from DTACPN-BN alone (*p* < 0.05).

Sling muscle strips exhibited greater contraction forces than the clasp muscle strips. The maximal contraction forces that were induced by GRP on the sling muscle strips were one and a half times greater than the clasp muscle strips, and the maximal contraction forces induced by DTACPN-BN on the sling muscle strips were two times greater than the clasp muscle strips (Fig. 2). Therefore, we used the sling muscle strips to investigate the mechanism underlying the GRP-induced contractions. The muscle strips were pretreated with TTX, atropine, and nifedipine. As shown in Fig. 4A, the GRP-induced dose-dependent contraction was almost unaffected by TTX (*p* > 0.05, compared with GRP alone, n = 4). Similarly, atropine had no significant effect on the GRP-induced contraction (*p* > 0.05, compared with GRP alone, n = 4). In contrast, the GRP-induced contraction of the LES sling muscle fibers was significantly inhibited by the L-type Ca^2+^ channel blocker, nifedipine (*p* < 0.05, compared with GRP alone, n = 4), at a concentration of 1 μM.

### The effects of TTX and ω-conotoxin GVIA (CTX) on DTACPN-BN-induced porcine LES sling muscle strip contractions

As shown in Fig. 4B, dose-dependent DTACPN-BN-induced contraction of the porcine LES sling muscle strips was significantly inhibited by either TTX or CTX (both *p* < 0.05, compared with DTACPN-BN alone, n = 4).

### Reverse transcription polymerase chain reaction (RT-PCR) analysis of BB_1_, BB_2_, and BB_3_ transcript levels in the LES sling and clasp muscle fibers

**Figure 5:**
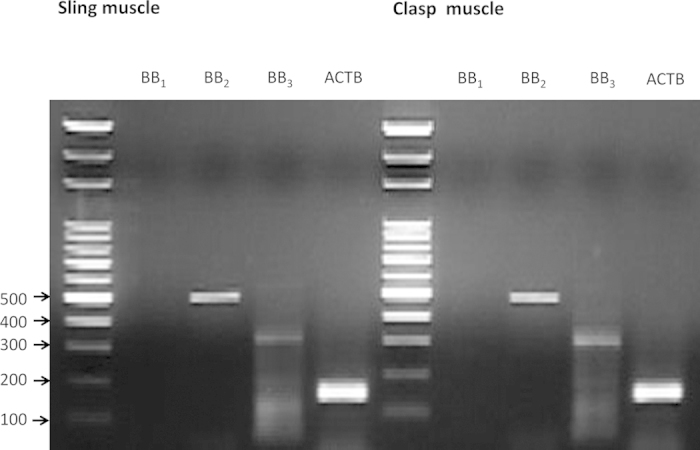
Reverse transcription polymerase chain reaction analysis of bombesin receptor mRNA expression in porcine LES sling and clasp muscles. Total RNA was reverse transcribed and amplified with bombesin receptor subtype 1 (BB_1_), 2 (BB_2_), 3 (BB_3_) and β-actin specific primers. The amplified products were electrophoresed on an agarose gel, stained with ethidium bromide and analyzed under UV light. The presented results are representative of three experiments. The BB_2_ and BB_3_ (lane BB_2_, 487 bp, and lane BB_3_, 373 bp, respectively) PCR products were detected in both the sling and clasp muscles. No BB_1_-specific PCR product was detected (lane BB_1_). The size of the β-actin specific PCR product, which was utilized as the internal control, was 148 bp (lane ACTB).

Using BB_2_-specific primers, we could generate a PCR product of 487 bp (Fig. 5) from both the sling and clasp muscle fibers. Likewise, a BB_3_-specific PCR product of 373 bp was obtained from both the sling and clasp muscle fibers. The size of the β-actin PCR product (the internal control) was 148 bp, as predicted. No PCR product corresponding to the NMB receptor (BB_1_) was detected in the cDNA that was generated from sling or clasp muscle fibers (n = 3).

### Immunohistochemistry (IHC)

**Figure 6:**
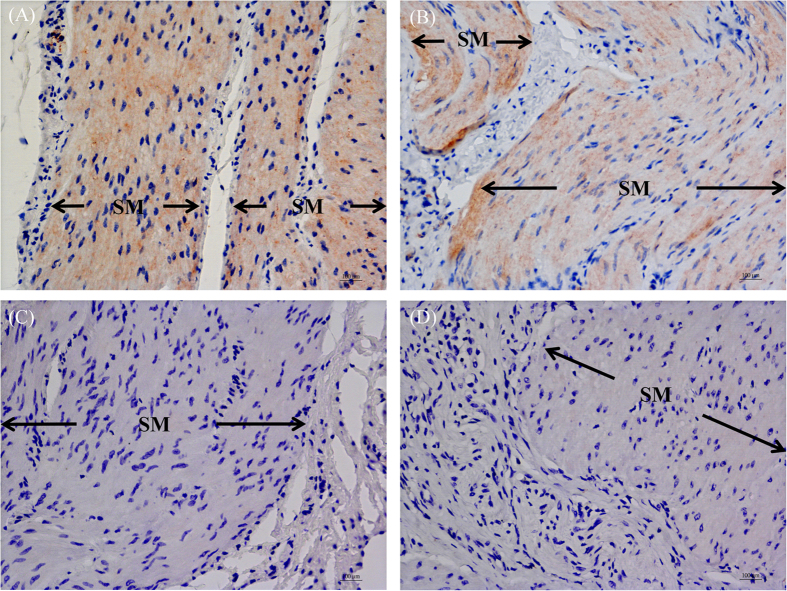
Immunohistochemical analysis of paraffin-embedded sling and clasp muscle fibers that were stained with bombesin receptor subtype 2 (BB_2_) specific antibodies. BB_2_ immunostaining was observed in the smooth muscle (SM) of LES sling (**A**) and clasp (**B**) muscles (magnification 400×). The sections were stained with equimolar concentrations of normal rabbit IgG, which was used as a negative control for the sling (**C**) and clasp (**D**) muscle staining (magnification 400×).

**Figure 7:**
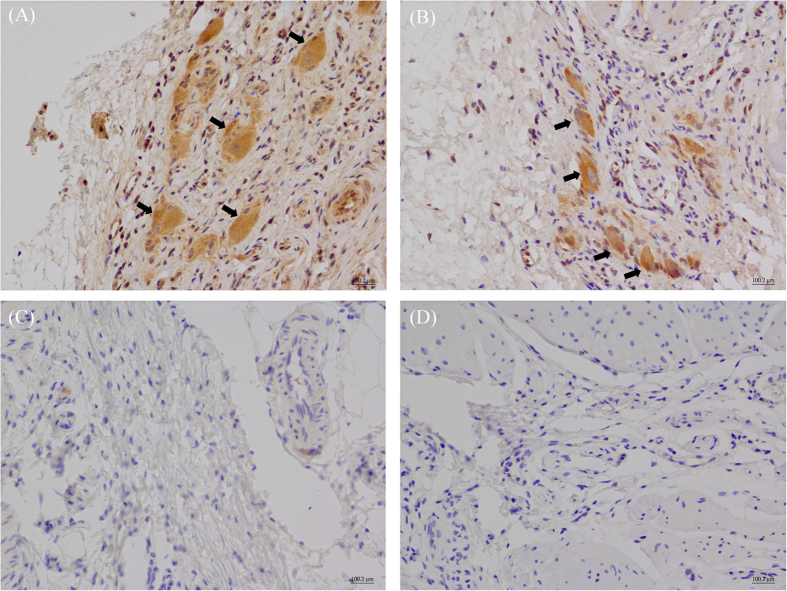
Immunohistochemical analysis of the paraffin-embedded sling and clasp muscle fibers stained with bombesin receptor subtype 3 (BB_3_) specific antibodies. BB_3_ immunostaining (arrows) was detected in the LES sling (**A**) and clasp (**B**) muscle ganglia (magnification 400×). The sections were stained with equimolar concentrations of normal rabbit IgG, which was used as a negative control for the sling (**C**) and clasp (**D**) muscle staining (magnification 400×).

Immunohistostaining of the neuromedin B receptor (BB_1_) with a specific BB_1_ antibody showed negative results in both the sling and clasp muscles. However, there was positive BB_1_ immunostaining on the positive control tissues of Sprague Dawley rat testes (as shown in Supplementary Fig. S1). In contrast, IHC results revealed that the GRP receptor (BB_2_) was ubiquitous in the LES sling (Fig. 6A) and clasp (Fig. 6B) muscle fibers (n = 3). In addition, immunosignals that were specific for the BB_3_ were found in myenteric ganglia of the LES sling (Fig. 7A) and clasp (Fig. 7B) muscles (n = 3).

## Discussion

Bombesin receptors are a family of G protein-coupled receptors, including the NMB receptor (BB_1_) as well as GRP receptors (BB_2_) and BB_3_. BB_2_ is widely present in the pancreas, central nervous system, and gastrointestinal tract. BB_2_ plays a role in many gastrointestinal functions, including gastrointestinal motility regulation, insulin stimulation, pancreatic and gastric acid secretion, colonic ion transport stimulation and the secretion of various hormones^15^. GRP, a BB_2_ agonist, plays a major role in gastric emptying, small bowel transit, and gallbladder contraction^16^. In this study, we showed that GRP can induce LES sling and clasp muscle fiber contractions in pigs and that the selective BB_2_ antagonist, DPDM-BN EA, can significantly inhibit GRP-induced LES contraction, indicating that GRP causes LES contraction via BB_2_.

The contraction of porcine LES smooth muscle fibers is modulated by multiple factors, especially autonomic neural innervations. The excitatory postganglionic vagal nerves transmit signals to nerve endings. Acetylcholine is released from the presynaptic nerve endings and stimulates postsynaptic muscarinic receptors. After stimulation of the muscarinic receptor, extracellular Ca^2+^ flows into the intracellular space, principally through L-type Ca^2+^ channels. Increased intracellular Ca^2+^ binds to calmodulin, which subsequently leads to Ca^2+^/calmodulin-dependent activation of myosin light chain kinase (MCLK), which in turn induces smooth muscle contraction^17^.

In the first step, we used TTX, a selective neuronal Na^+^ channel blocker, to explore whether GRP acts on the nerve fibers or directly on the LES smooth muscle. We found that TTX did not inhibit GRP-induced contraction of the LES, suggesting that the contraction was not due to an effect of GRP on the LES neuronal fibers. Additionally, atropine, a nonselective muscarinic receptor antagonist, was applied to investigate whether GRP acts directly on the muscarinic receptor of the LES smooth muscle, resulting in contraction. The GRP-induced contraction was not inhibited by atropine, indicating that GRP does not affect the LES muscarinic receptors. In contrast, we found that nifedipine could block GRP-induced LES contractions. Nifedipine is an L-type Ca^2+^ channel blocker that inhibits extracellular Ca^2+^ influx, and LES smooth muscle contraction is generally related to an increase in the intracellular Ca^2+^ concentration^18^. Our results show that the extracellular Ca^2+^ influx through the L-type Ca^2+^ channel was indeed a main GRP-induced contraction pathway of the porcine LES. However, the L-type calcium blocker may also decrease the intracellular calcium stores in muscle strips owing to blockade of calcium influx which is needed for calcium store refilling. Therefore, the role of intracellular calcium stores cannot be excluded completely.

BB_3_ was first cloned from the guinea pig uterus^19^. BB_3_ plays a role in the regulation of energy homeostasis, glucose/insulin levels, lung development, satiety, and injury^15^. Previous studies showed that BB_3_ is present in the myenteric and submucosal ganglia in the tunica muscularis of the gastrointestinal tract and in nerve fibers between the myenteric ganglia. Additionally, with specific BB_3_ antibodies, it was also detected in interstitial cells of Cajal (ICC)^20^. To date, the function of BB_3_ in the gastrointestinal tract is not clear, and it was proposed that BB_3_ might be related to gastrointestinal motility regulation^20^. In this study, we showed that the selective BB_3_ agonist, DTACPN-BN, could induce LES sling and clasp muscle fiber contractions in pigs and that the selective BB_3_ antagonist, Bantag-1, could significantly decrease porcine LES sling and clasp muscle fiber contractions, suggesting that DTACPN-BN causes LES contraction via BB_3_.

The neuronal Na^+^ channel blocker, TTX, and the neuronal Ca^2+^ channel blocker, CTX, were used to explore the mechanism of BB_3_ agonist-induced LES contraction in pigs. Our results revealed that both TTX and CTX significantly decreased BB_3_ agonist-induced contraction of the porcine LES sling muscle fibers. This finding implies that nerve conduction was associated with the BB_3_ agonist-induced LES contraction.

RT-PCR identified BB_2_ and BB_3_ transcripts in the LES sling and clasp muscles; however, BB_1_ transcripts were not detected. This finding is in good agreement with the results we obtained for the BB_2_ and BB_3_ agonist-induced LES sling and clasp muscle fiber contractions. Immunohistochemistry with specific BB_2_ and BB_3_ antibodies showed that BB_2_ was mainly expressed in the muscle, while BB_3_ was principally present in the ganglia. Taken together, these findings reveal that BB_2_ and BB_3_ are localized in the LES sling and clasp muscles in pigs at both the mRNA and protein levels.

Clinically, proton pump inhibitor refractory GERD can be non-reflux or reflux related. The reflux-related causes include residual acid reflux due to inappropriate medical treatment or poor compliance, non-acid reflux such as duodeno-gastro-esophageal (bile) reflux, hypersensitivity to physiological amounts of acid, and persistent impairment of esophageal mucosal integrity^2^. We used selective bombesin receptor agonists and antagonists to demonstrate that both BB_2_ and BB_3_, but not BB_1_ agonists, could cause tonic LES contraction in a dose-dependent manner. Therefore, the BB_2_ and BB_3_ receptors were both involved in the bombesin-induced LES contraction. These results support the hypothesis that bombesin and its agonists are a new kind of potential drug for the enhancement of esophageal sphincter contraction in order to avoid irritant reflux. These BB_2_ and BB_3_ agonists will be helpful to treat or as adjuvant therapy for patients with proton pump inhibitor refractory GERD or those suffering from the side effects of long-term PPI use.

In conclusion, the present study showed that bombesin-induced LES contraction occurs through BB_2_ and BB_3_. This study also indicates that BB_2_-mediated contraction is not due to the action of nerve and muscarinic receptors, but rather is caused by the influx of extracellular Ca^2+^ into myocytes through L-type Ca^2+^ channels. In contrast, the BB_3_-mediated tonic LES contraction was associated with neuronal conduction. Therefore, bombesin, as well as BB_2_ and BB_3_ agonists, might have the potential to treat GERD.

## Materials and Methods

Porcine stomach, including the lower esophagus, was purchased from a local slaughterhouse. The stomachs, from pigs weighing approximately 110 Kg, were placed in ice-cold Kreb-Henseleit buffer solution that had the following composition (in mM): 15.5 NaHCO_3_, 122 NaCl, 4.7 KCl, 1.2 KH_2_PO_4_, 1.2 MgCl_2_, 1.8 CaCl_2_, 11.5 glucose, pH 7.4. Additionally, the stomachs were kept on ice during transport to the laboratory, which lasted approximately 30 minutes. Carbachol, atropine, nifedipine, ω-conotoxin GVIA (CTX), Bantag-1 (BB_3_ antagonist), and buffer reagents were obtained from Sigma-Aldrich, MO, USA. Tetrodotoxin (TTX) was purchased from Tocris Bioscience, Bristol, UK. Bombesin, neuromedin B (NMB), gastrin-releasing peptide (GRP), and [D-Phe^6^,des-Met^14^]BN(6-14) ethylamide (DPDM-BN EA, BB_2_ antagonist) were obtained from the American Peptide Company, CA, USA. [D-Tyr^6^,Apa-4Cl^11^,Phe^13^,Nle^14^]bombesin-(6-14) (DTACPN-BN, BB_3_ agonist) was purchased from Phoenix Pharmaceuticals, Burlingame, CA, USA. PD 168368 (BB_1_ antagonist) was purchased from Santa Cruz Biotechnology, Dallas, Texas, USA. These agonists and antagonists were chosen because of the specificity and high affinity for the respective bombesin receptor subtypes^15^,^21^.

### The effects of bombesin, NMB, GRP, and DTACPN-BN on porcine LES sling and clasp muscle tension

The porcine LES is often used as an animal model to study esophageal motility and GERD^22^,^23^. Fresh porcine esophagus and stomachs were cut longitudinally along a midline between the GC and LC of the stomach. The mucosa was removed with surgical scissors. The bigger semicircular sling and smaller semicircular clasp muscle bundles of the LES were identified and obtained as previously described^24^. The muscle strips, which were 3 mm wide and 10 mm long, were trimmed from the sling and clasp muscles, respectively, and suspended in 7 ml organ baths containing Krebs-Henseleit buffer solution, incubated at 37 °C, and continuously gassed with 95% O_2_ + 5% CO_2_. Subsequently, the muscle strips were connected to isometric force transducers (FORT10g, World Precision Instruments Inc., Sarasota, FL, USA), which were connected to amplifiers and a computer recording system (BIOPAC Systems, CA, USA). The basal tension of the muscle strips was set at 1.0 g. After a 30 min equilibration period, carbachol (1 μM) was added to the organ bath, the muscle strip contractions were measured, and the carbachol was washed out. Carbachol-induced contraction served as a reference (100%) for the contractile response to bombesin or the selective agonists^25^. After another equilibration period, bombesin, NMB, GRP, or DTACPN-BN was added to the organ bath in a cumulative dose (1 nM, 10 nM, 100 nM, 1 μM, and 3 μM), and the isolated sling or clasp sphincter muscle strip contractions were measured.

### The effects of PD 168368, DPDM-BN EA and Bantag-1 on the porcine LES sling and clasp muscle strip tension

PD 168368 is a selective BB_1_ antagonist. DPDM-BN EA is a selective BB_2_ antagonist, and Bantag-1 is a selective BB_3_ antagonist. To investigate whether BB_2_ is involved in bombesin-induced LES contraction, the isolated sling or clasp muscle strips were treated with DPDM-BN EA (1 μM) for 6 min, followed by the addition of GRP to the organ bath in a cumulative manner^26^. Similarly, to investigate whether BB_3_ is involved in bombesin-induced LES contraction, the isolated sling or clasp muscle strips were treated with Bantag-1 (1 μM) for 6 min, followed by the addition of DTACPN-BN to the organ bath in a cumulative manner^21^. In addition, to investigate which receptor is involved in bombesin-induced LES contraction, the isolated sling or clasp muscle strips were treated with PD 168368, DPDM-BN EA, Bantag-1 (1 μM respectively) or DPDM-BN EA combining with Bantag-1 (both 1 μM) for 6 min, followed by the addition of bombesin to the organ bath in a cumulative manner.

### The effects of TTX, CTX, atropine, and nifedipine on GRP- or DTACPN-BN-induced porcine LES sling muscle strip contractions

TTX, atropine, and nifedipine are neuronal Na^+^ channel, muscarinic receptor, and L-type Ca^2+^ channel blockers, respectively. To investigate the mechanism of GRP- or DTACPN-BN-induced LES contraction in the pigs, the isolated sling muscle strips were treated with TTX (1 μM, 15 min), atropine (1 μM, 6 min), or nifedipine (1 μM, 20 min). GRP or DTACPN-BN was added to the muscles strips that were treated with TTX. Additionally, GRP was added to the muscle strips that were treated with either atropine or nifedipine. Furthermore, the neuronal Ca^+^ channel blocker, CTX (1 μM), was used to pretreat the isolated sling muscle strips, and 15 min later, DTACPN-BN was added to the organ bath^27^,^28^,^29^.

### RT-PCR for detection of bombesin receptor mRNA in porcine LES

Sling and clasp muscle fibers that were obtained from the porcine LES were stored in RNAlater solution (Applied Biosystems Inc., Foster City, CA, USA) at 4 °C for two days to allow for tissue penetration, and then, excess liquid was removed and the tissue was stored at −80 °C until use.

RNA was isolated from tissue samples with the guanidine isothiocyanate method, using the GeneJET RNA Purification Kit (Thermo Fisher Scientific Inc., Waltham, USA), according to the manufacturer’s protocol. The quality and purity of the isolated RNA were measured with an UV/Vis spectrophotometer (DU800, Beckman Coulter, CA, USA).

After RNA concentration measurements, cDNA was synthesized using the extracted RNA and the High Capacity cDNA Reverse Transcription Kit (Applied Biosystems Inc., CA, USA) for RT-PCR. The RNA sample preparation for reverse transcription was performed according to the manufacturer’s recommendation. A volume corresponding to 1 μg of RNA was used for the cDNA synthesis. The cDNA samples were diluted to a volume of 100 μl and prepared for subsequent PCR.

Amplification of the bombesin receptor-specific sequences through PCR was carried out on a thermal cycler (Thermo PX2, MA, USA) with the All-in-One PCR Mix (Protech, Taipei, Taiwan) under the following program: one cycle of 95 °C for 10 min, 35 cycles of 95 °C for 30 s, 53 °C for 50 s, and 72 °C for 30 s, and one cycle of 72 °C for 10 min. As an internal control, ß-actin was amplified. The primer sequences were the following: (1) β-actin: forward 5′-TCGGTTGGATGGAGCATCCCC-3′ and reverse 5′-GGGAAGGCAGGGACTTCCTGTAA-3′; (2) neuromedin B receptor (BB_1_): forward 5′-GCGGACAGGTACAGAGCAAT-3′ and reverse 5′-GGCGTTTCCGTGTTTCCATC-3′; (3) gastrin-releasing peptide receptor (BB_2_): forward 5′-CGTGCACTGCCACATCTCTA-3′ and reverse 5′-AAACACAGCCTCTGGGATGG-3′; (4) bombesin-like receptor 3 (BB_3_): forward 5′-AACGCCATCCTGAAGACCTG-3′ and reverse 5′-AGAGAGCAAACAGAGCCACC-3′. The PCR products were separated by electrophoresis in a 2% agarose gel, which was then stained with ethidium bromide and visualized under UV light.

### Immunohistochemistry

Formalin-fixed and paraffin-embedded porcine sling and clasp muscle fibers were prepared for IHC staining. The IHC stains were performed using standard reagents and techniques on a BOND-MAX Automated Staining System (Leica Microsystems). Briefly, the deparaffinized and rehydrated sections were subjected to heat-induced antigen restoration with bond epitope retrieval solution 1 (citrate-based pH 6.0 solution, Leica Microsystems). The staining procedure involved a peroxidase block with 3% hydrogen peroxide for 5 min, incubation with a 1:300 dilution of rabbit neuromedin B receptor polyclonal antibody, LS-A825 (Lifespan Biosciences, Seattle, WA, USA) for BB_1_, a 1:300 dilution of rabbit GRPR polyclonal antibody (Abnova, Taipei, Taiwan) for BB_2_, or a 1:200 dilution of rabbit anti-Brs3/bombesin receptor 3 polyclonal antibody (Bioss, MA, USA) for BB_3_ for 30 min at room temperature. Then, the samples were subsequently incubated with an anti-rabbit horseradish peroxidase polymer for 10 min at room temperature. The samples were then treated with a chromogen, 3,3′-diaminobenzidine tetrahydrochloride (DAB), for 10 min at room temperature and counterstained with hematoxylin for 5 min. The sling and clasp muscle sections were stained with normal rabbit IgG at equimolar concentrations as negative controls.

### Data analysis

These data are expressed as the mean ± standard error of the mean (SEM). GraphPad Prism 5 was used to determine the half-maximal contraction (EC_50_) values. The statistical analysis of the results was performed with Student’s *t*-tests or one-way ANOVA followed by Tukey’s *post hoc* test. In all cases, the differences were considered significant when *p* < 0.05.

## Additional Information

**How to cite this article**: Tsai, C.C. *et al.* Mechanism of bombesin-induced tonic contraction of the porcine lower esophageal sphincter. *Sci. Rep.* **5**, 15879; doi: 10.1038/srep15879 (2015).

## Supplementary Material

Supplementary Information
