# Supplementary material for: Mechanism of bombesin-induced tonic contraction of the porcine lower esophageal sphincter
Source: Sci Rep. 2015 Nov 2;5:15879. doi: 10.1038/srep15879 (PMC4629149; doi:10.1038/srep15879)
Supplement: Supplementary Information [file srep15879-s1.pdf]

Mechanism of bombesin-induced tonic contraction of the porcine lower esophageal sphincter

Ching-Chung Tsai , Li-Ching Chang, Kai-Jen Lin, Shu-Leei Tey, Yu-Tsun Su,

Ching-Wen Liu, Tong-Rong Tsai, Shih-Che Huang

A

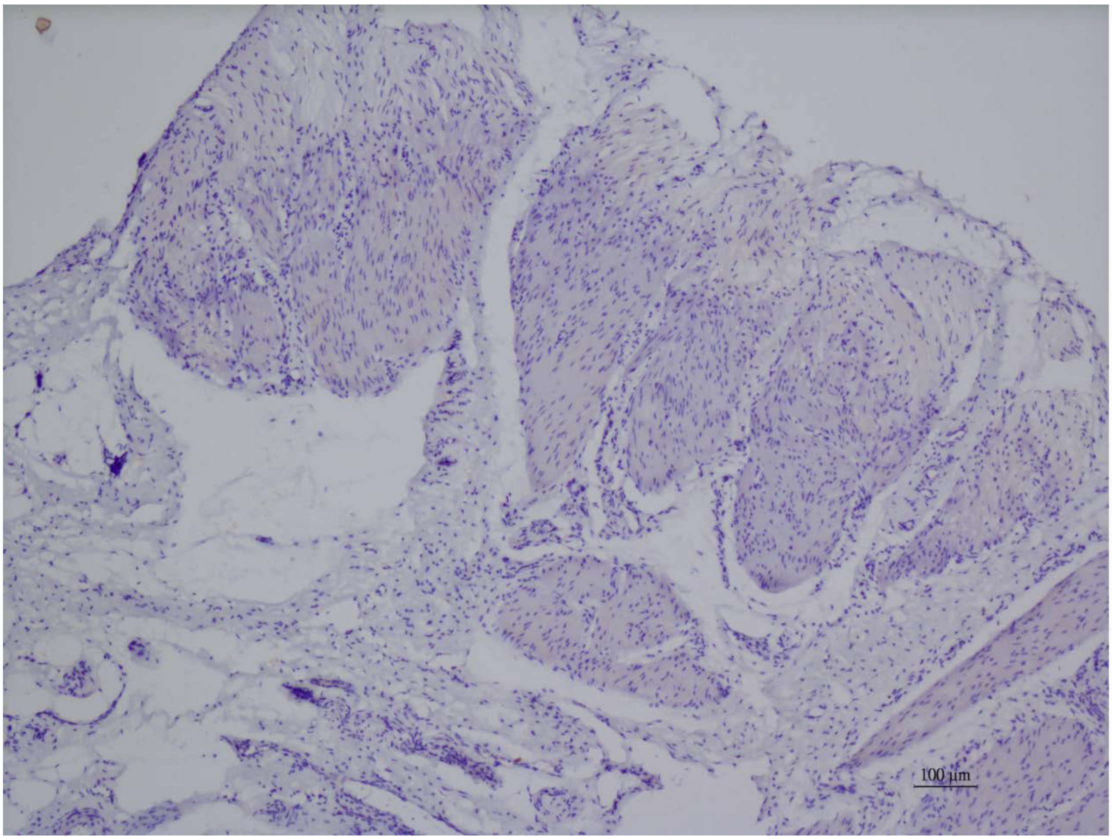

B

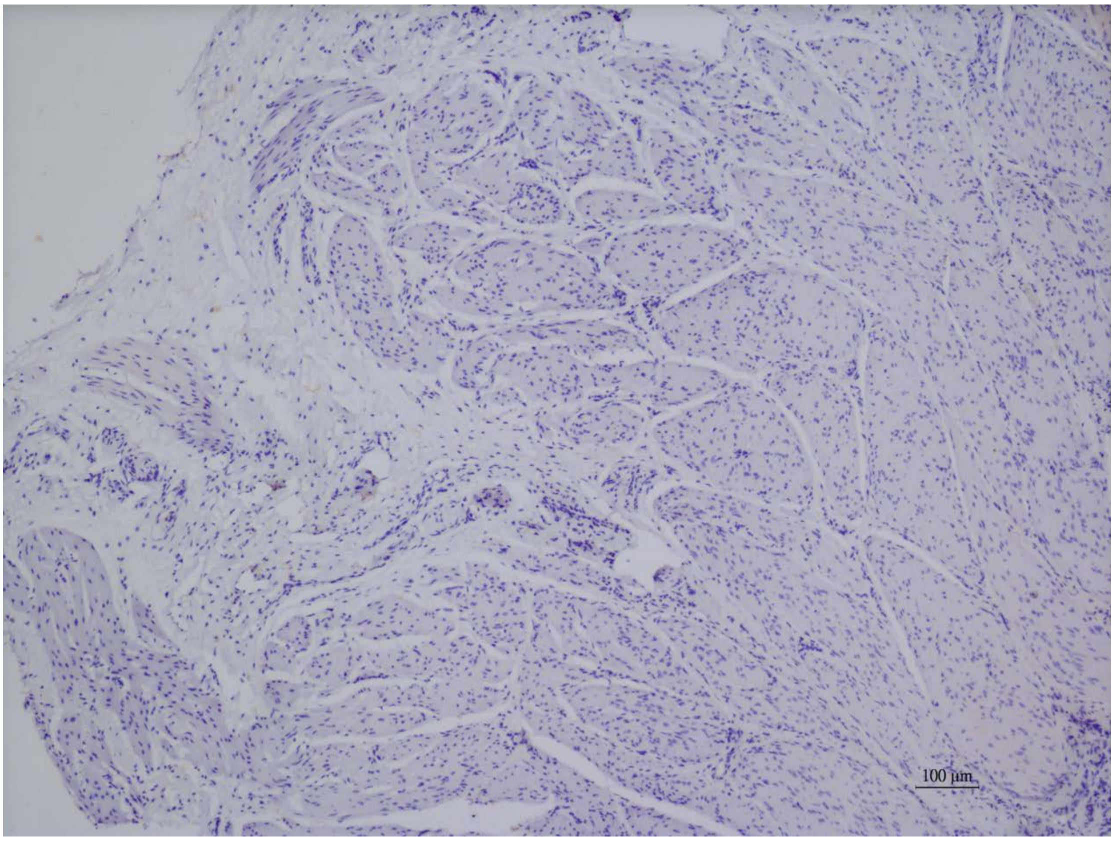

C

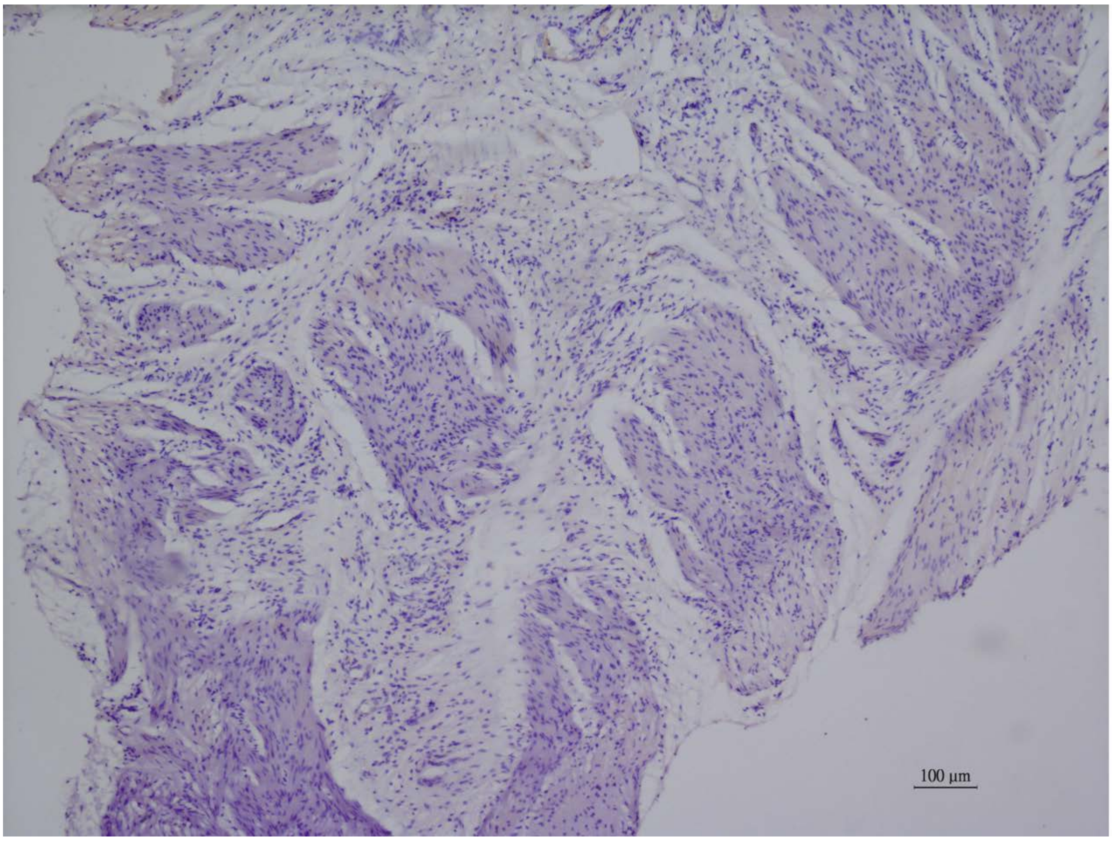

D

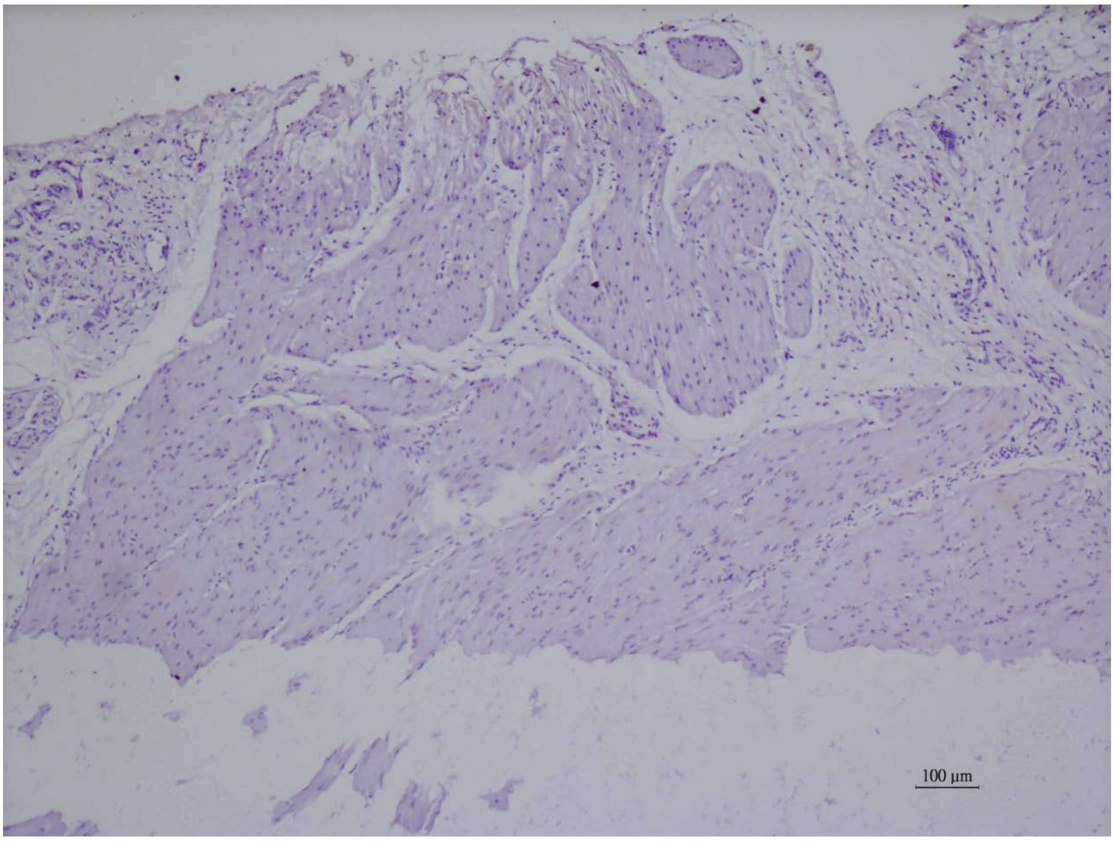

E

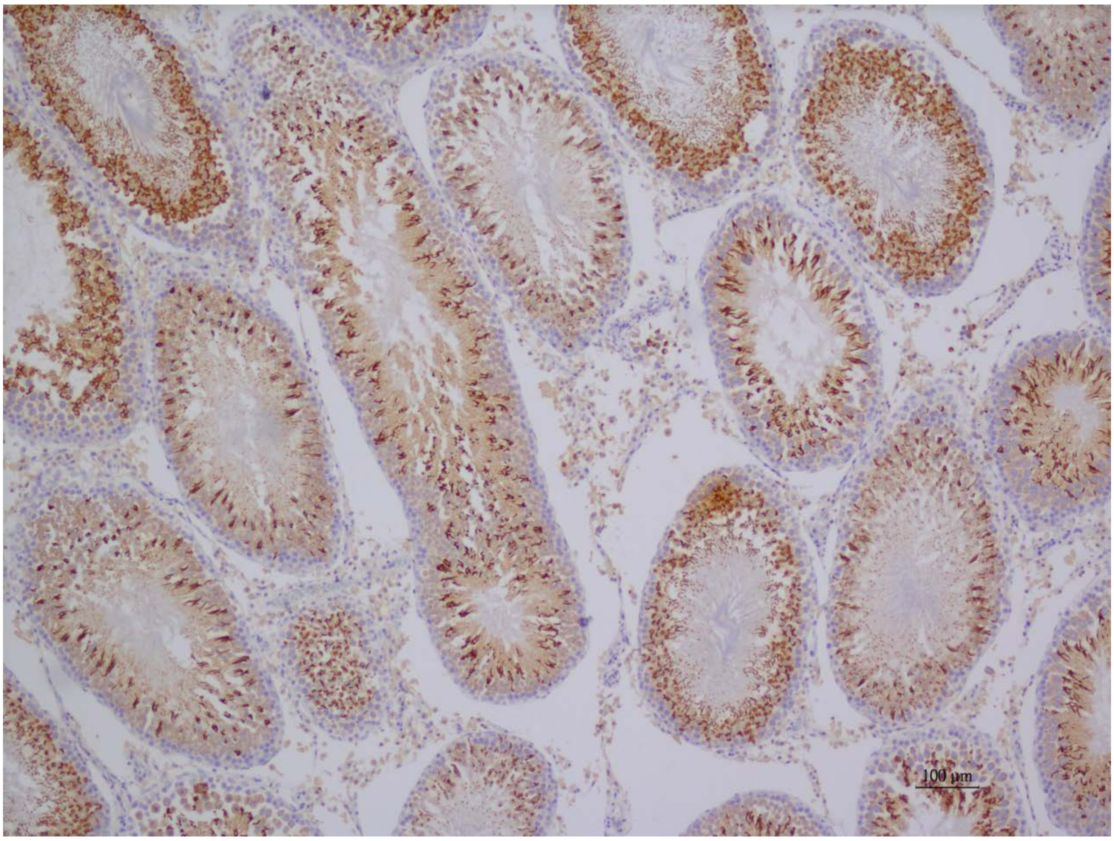

F

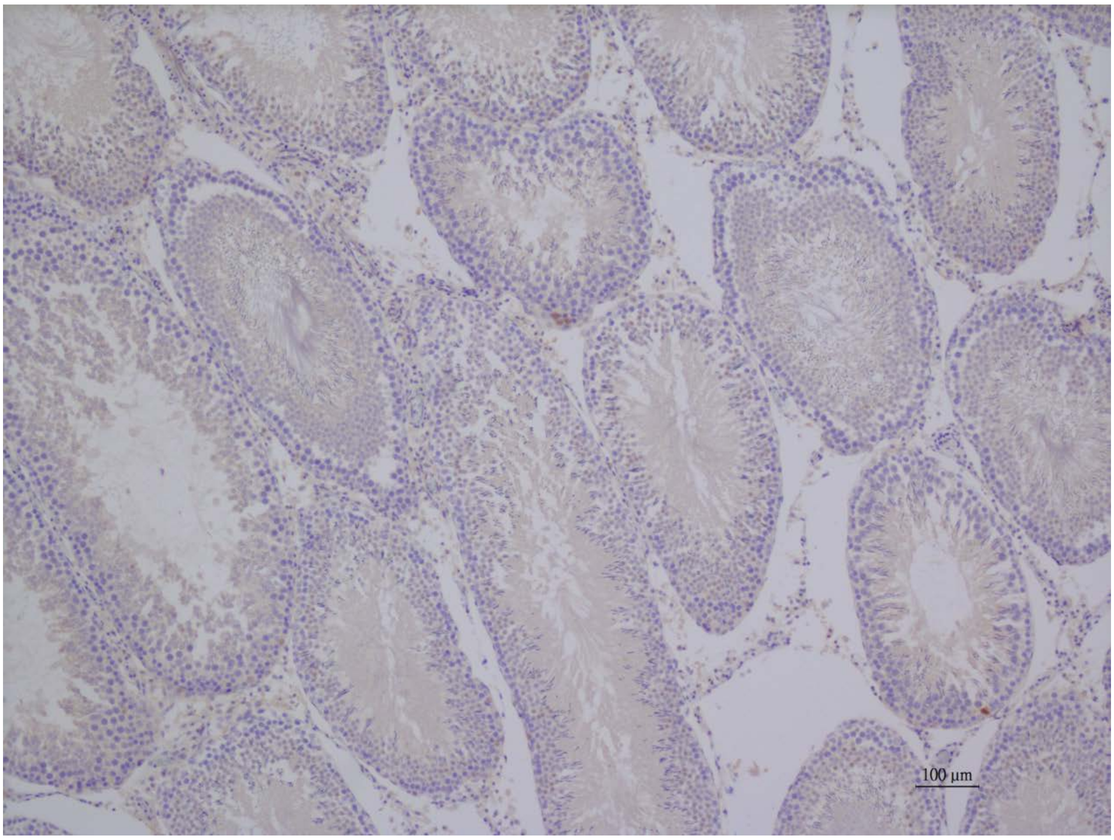

Supplementary Figure S1. Immunohistochemical analysis of the paraffin-embedded sling and clasp muscle fibers stained with a bombesin receptor subtype 1 (BB_1) specific antibody and BB_1 immunostaining was not detected in the LES sling (A) and clasp (B) muscle strips (n = 3, magnification 100×). The tissue sections were stained with equimolar concentrations of normal rabbit IgG, which was used as a negative control for the sling (C) and clasp (D) muscle staining (magnification 100×). BB_1 immunostaining was detected in the rat testes (E) and the sections were stained with equimolar concentrations of normal rabbit IgG, which was used as a negative control for rat testes (F) (magnification 100×).
